# Parameters influencing *Agrobacterium*-mediated transformation system in safflower genotypes AKS-207 and PKV Pink

**DOI:** 10.1007/s13205-016-0497-4

**Published:** 2016-08-26

**Authors:** Dipti Raghunath Dhumale, Prashant Raghunath Shingote, Mahendra Shankarrao Dudhare, Pravin Vishwanath Jadhav, Prashant Bhaskar Kale

**Affiliations:** 1 Department of Agricultural Botany, Biotechnology Centre, Dr Panjabrao Deshmukh Agricultural University, Akola, MS 444104 India; 2 National Research Centre on Plant Biotechnology, Pusa Campus, New Delhi, 110012 India

**Keywords:** Indirect regeneration, *Agrobacterium tumefaciens*, GUS histochemical assay, *Carthamus tinctorius* L., Oilseed

## Abstract

Shoot regeneration in safflower (*Carthamus tinctorius* ‘AKS 207’ and ‘PKV Pink’) genetically transformed using *Agrobacterium* was used for assessing various constraints to the efficiency of transformation including infection period, virulence induction medium, co-cultivation period, bacterial titre, selection regime, and the natural phenolic compound acetosyringone. Transformation frequency was promising with 8–10-day-old cotyledonary leaf explants. Therefore, explants of that age cultured on *Agrobacterium* minimal medium (AB) containing 100 µM acetosyringone were infected with *Agrobacterium* (cell titre 0.5 OD_600nm_) for 15 min followed by 48 h of co-cultivation on kanamycin-enriched medium (50 mg/L). Transformation of the shoots was confirmed using β-glucuronidase (GUS) histochemical assay and polymerase chain reaction (PCR). With the transformation protocol thus optimized, the transformation frequency as determined using GUS assays was 54.0 % for AKS 207 and 47.6 % for PKV Pink. The corresponding figures using PCR were 27.0 and 33.3 %. The transformed shoots required 10–14 weeks of culture initiation but produced very few roots.

**Electronic supplementary material:**

The online version of this article (doi:10.1007/s13205-016-0497-4) contains supplementary material, which is available to authorized users.

## Introduction

Safflower (*Carthamus tinctorius*), family Asteraceae (Compositae), is annually cultivated in the tropics and subtropics of the world. It is cultivated mainly for its seeds and flowers which have commercial values (Vijaya Kumar et al. 2008). The flowers are useful in medicine, as a source of a colouring agent and of fibres, and for food flavouring. Safflower oil is rich in linoleic acid (75–90 %) and is believed to lower cholesterol levels in blood. Safflower is, therefore, important in food, pharmaceutical, paint, and lubricant industries (Lijiao and Meili 2013). Safflower oil commands a higher price than other edible oils, but the higher price is offset by the potential health benefits of the oil (Li and Mündel 1996). Safflower is particularly suited for molecular farming and is used in the production of human insulin, lipoproteins, growth hormones, and specialty oils of high nutritional value (Shilpa et al. 2010). A major drawback of safflower is that it is susceptible to insect pests. The safflower aphid (*Uroleucon compositae*) is the most serious pest, which is estimated to reduce yields by 30–80 % (Hanumantharaya et al. 2008); at the same time, the existing measures for pest control are expensive, and various management practices and breeding strategies are not particularly effective.

Genetic engineering offers some advantages over traditional methods of breeding, and *Agrobacterium*-meditated transformation of genes has become a gold standard (Shingote et al. 2015; Kharte et al. 2016), a method that integrates fewer copies of trans genes into plants compared to the biolistic method and is, therefore, the preferred method for obtaining stable expression of trans genes and avoiding trans gene silencing (Joyce et al. 2010; Ramesh et al. 2004). In this process, only the T-DNA region of the vector is transferred, a region engineered to encompass a selectable marker, a reporter gene, and the genes of interest, which are then transferred from the bacterium to the host plant’s nuclear genome. These functions are facilitated by a set of Virulence (*Vir*) genes in the presence of acetosyringone (AS), a phenolic inducer released by wounded plant cells (Ali et al. 2007).

Safflower, however, has not proved amenable to genetic manipulation: in vitro regeneration has proved difficult; and *Agrobacterium*-mediated transformation has been tried in only a few varieties of safflower (Ying et al. 1992; Nikam and Shitole 1999; Rao and Rohini 1999; Rohini and Rao 2000; Belide et al. 2011; Motamedi et al. 2011). The present work, therefore, examines some of the transformation parameters in detail to find out ways to deploy genetic transformation in two safflower genotypes, namely AKS 207 and PKV Pink.

## Materials and methods

### Tissue culture

Certified seeds of safflower genotypes AKS 207 and PKV Pink were obtained from the Oilseeds Research Unit, Dr Punjabrao Deshmukh Agricultural University, Akola, Maharashtra, India, and germinated in vitro. Cotyledonary leaf explants (about 0.5–1 cm^2^) were excised from 8- to 10-day-old seedlings and placed in the callus induction medium (CIM), which was nothing but Murashige and Skoog (MS) Medium (Murashige and Skoog 1962) supplemented with 2,4-dichlorophenoxyacetic acid and kinetin. The calli were induced and maintained on a mix containing one part of MS medium mixed with one, two, or three parts of CIM. Shoot induction from embryogenic calli was carried out on different combinations of the shoot induction medium (SIM), which contained MS medium supplemented with BAP (1, 2, 3, 4 or 5 mg/L), alone or in combination with naphthalene acetic acid (0.5 mg/L). Rooting of the induced multiple shoots was attempted on half-strength MS medium supplemented with different hormonal combinations reported in the literature and with a few more combinations devised for the present experiment.

### Bacterial strain, vector construct, and culture conditions

**Fig. 1:**
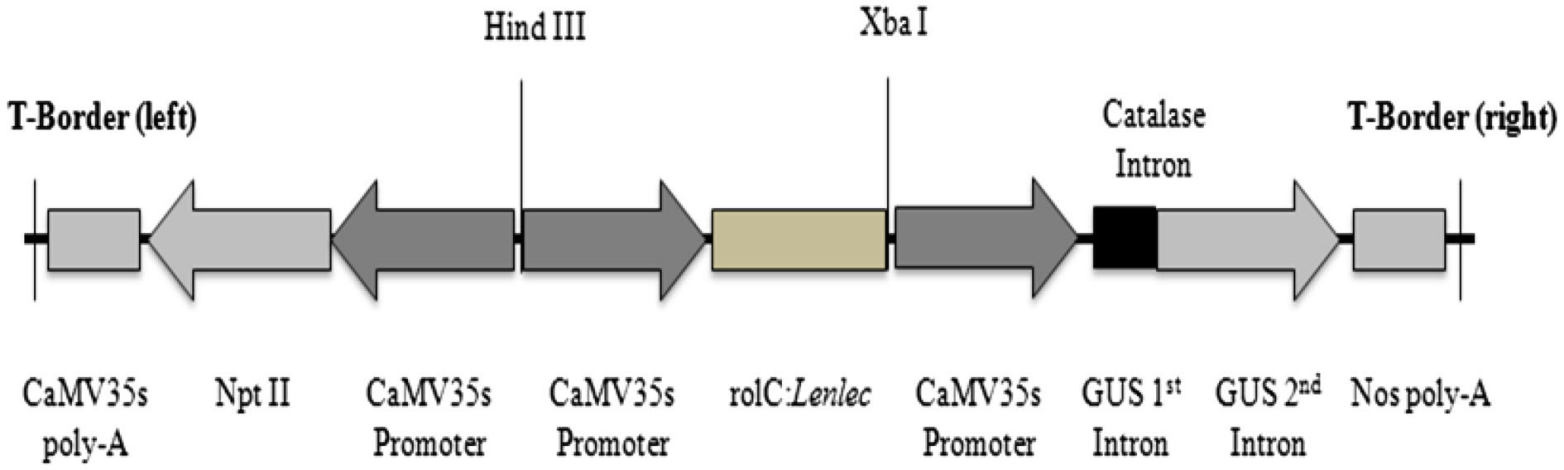
Recombinant pCambia2301::*Lentil*-*lectin* gene construct for safflower transformation

*Agrobacterium* strain EHA-105 harbouring the recombinant plasmid pCAMBIA2301 (CAMBIA, Canberra, Australia) was used in the safflower transformation system (Fig. 1). The bacteria were grown aseptically in Luria–Bertani (LB) medium containing two antibiotics, namely kanamycin (50 mg/L) and rifampicin (10 mg/L), 28 °C.

### Sensitivity to kanamycin

Because the vector construct harboured *npt*II gene as the plant selection marker, kanamycin was used as the selection agent. To determine the effective dose of kanamycin, the explants were cultured in optimized CIM supplemented with different concentrations of kanamycin (0, 25, 50, 75 and 100 mg/L) in Petri dishes, and kanamycin-free CIM was used as a control (Sujatha et al. 2012). The resulting calli were subcultured routinely every 2 weeks and observed closely every 3–4 days for necrosis. The percentage of explants showing necrosis was recorded after 4 weeks of incubation.

### *Agrobacterium* infection and co-cultivation

Cotyledonary leaf explants prepared as above were inoculated by exposing them for 20 min to *Agrobacterium* cultured on bacterial growth medium (BGM) enriched with AS. The explants were blotted dry and transferred to the optimized callusing medium supplemented with appropriate AS concentration and co-cultivated in dark at 26 ± 2 °C. After co-cultivation, the explants were washed with sterile distilled water alone and with 500 mg/L cefotaxime and transferred to MS medium supplemented with the optimized callusing and selection medium. After 10–14 weeks, the successfully regenerated and putatively transformed multiple shoots from calli were selected for further confirmation of gene integration.

For optimizing a genotype-independent transformation method for safflower, the cotyledonary leaf explants were cultured under different conditions. The deliberate variables included bacterial titre (0.25, 0.50, 0.75, and 1.0 OD at 600 nm) in combination with different lengths of the infection period (5, 10, 15, and 20 min), variations of the BGM (LB, AB, and MS), AS concentration (0, 50, 100, and 200 µM), and duration of assisted transformation (0, 24, 48 and 72 h). To enhance the penetration of the target tissues by *Agrobacterium*, the explants were pre-treated in individual experiments. Transformation efficiency was recorded as the number of putatively transformed multiple shoots obtained after 5–7 cycles of selection (10–14 weeks) (Sujatha et al. 2012).

### Histochemical analysis of gene expression

The antibiotic-resistant putatively transformed shoots were subjected to histochemical analysis (Jefferson 1987) of gene expression using GUS assays 10–14 weeks after *Agrobacterium* infection. Untransformed explants cultured under identical conditions served as controls (Shilpa et al. 2010). The cultures were checked visually for regenerating shoots and photographed after colour development and then examined for the presence of blue spots.

### Extraction of genomic DNA and confirmation using polymerase chain reaction

The integration of the *Lentil*-*lectin* gene with safflower genome of the putatively transformed GUS-positive multiple shoots was further confirmed through polymerase chain reaction (PCR). A modification of the Doyle and Doyle method that uses cetyltrimethyl ammonium bromide (CTAB) was used for isolating the DNA from putatively transformed and non-transformed (control) multiple shoots (Doyle and Doyle 1990). For PCR confirmation, a pair of *Lentil*-*lectin*-gene-specific primers was used. The recombinant plasmid served as a positive control, and DNA from the non-transformed plants served as a negative control. The amplified products were separated on 1.0 % (w/v) agarose gel using gel electrophoresis (Mini-Sub^®^ Bio-Rad, USA).

### Statistical analysis

Observations on the explants producing putatively transformed calli were recorded after 4 weeks of incubation for each selection cycle. Data were analysed using one-way analysis of variance (ANOVA). The mean values of treatments were subjected to Duncan’s multiple range test (DMRT) at 0.05 % level of significance and determined using SPSS ver. 11.09.

## Results

### Tissue culture

**Fig. 2:**
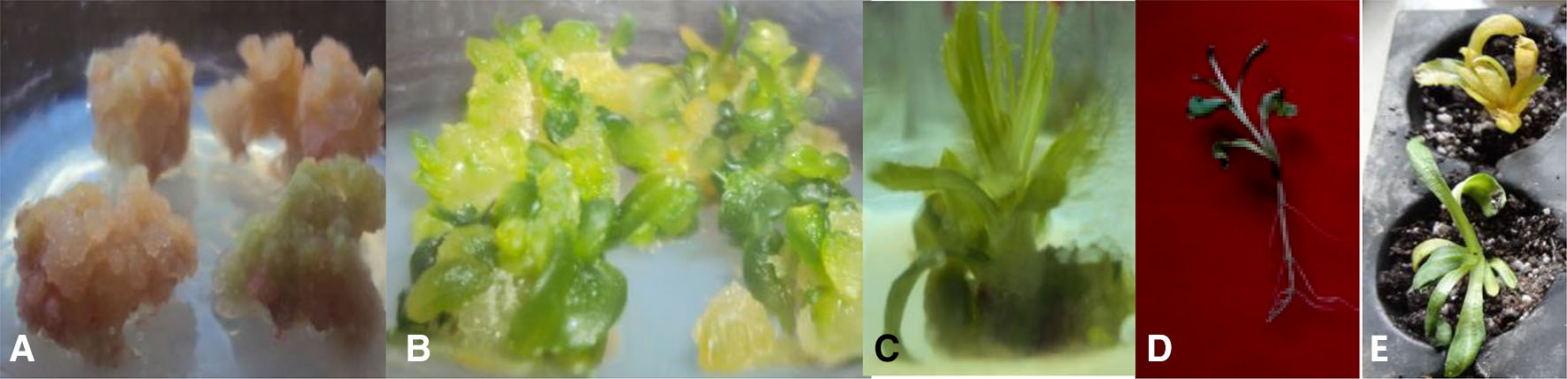
Optimized regeneration protocol for safflower genotypes AKS-207 and PKV pink. **a** Callus formation. **b** Shoot induction. **c** Multiple shoot formation. **d** Rooting. **e** Hardening in pro tray

Within 3–4 weeks, extremely fragile and pale yellow (embryogenic) calli appeared on MS medium supplemented with 1 mg/L each of 2–4, D and kinetin (Fig. S1). The callusing percentage was found to be maximum (100 % in AKS 207 and 99 % in PKV Pink) in that version of MS medium. When the ratio was changed to 2 or 3 parts of kinetin to 1 part of 2, 4-D, callusing in AKS 207 was 99 and 98 %, respectively, whereas in PKV Pink the corresponding figures were 98 and 97 %. When the calli were about 7–10 weeks old, they were transferred to SIM for inducing multiple shoots. In MS medium supplemented with BAP at 1, 2, 3, 4 and 5 mg/L, shoot induction percentages were, respectively, 40, 54, 67, 66 and 66 % in AKS 207 and 34, 40, 55, 65 and 61 % in PKV Pink (Fig. S2). Root induction was attempted in elongated multiple shoots employing various hormonal combinations as reported by Nikam and Shitole (1999) and by Mandal and Gupta (2001) but rooting 8 % in both the genotypes was observed only in MS supplemented with 2 mg/L NAA: all the other combinations failed to induce rooting. The regeneration of whole plant was complete within 12–13 weeks of culture initiation (Fig. 2).

### Sensitivity test for selecting transformed tissues

The transformed tissues proved sensitive to kanamycin: the greater the concentration of kanamycin, the higher the frequency of necrosis in the explants and the lower the percentage of callus induction and survival (Table 1). On the other hand, the explants grown on media without kanamycin (the control) showed higher survival and better growth. After 4 weeks of culture initiation, kanamycin at 25 mg/L led to 30.8 % explants showing necrosis in AKS 207 and 22.8 % in PKV Pink; when the concentration was increased to 50 mg/L, the LD_50_ of necrosis was 52 % in AKS 207 and 54 % in PKV Pink; even higher concentrations proved more damaging still, with 83.3 % of the explants in AKS 207 and 97.3 % of the explants in PKV Pink being necrosed at 75 mg/L. The corresponding figures were 80.7 and 98.7 % at 100 mg/L. At higher concentrations, the explants showed necrosis within 4–8 days of culture (Table 1).

**Table 1:** Sensitivity of safflower to kanamycin during somatic organogenesis through callus induction from cotyledonary leaf explant

Kanamycin mg/L	Necrosis in %	
	AKS-207	PKV PINK
0	9.33 ± 2.9e	6.00 ± 2.3e
25	30.67 ± 2.9d	22.67 ± 2.9d
50	52.00 ± 3.5c	54.00 ± 5.8c
75	83.33 ± 4.4b	80.67 ± 5.8b
100	97.33 ± 1.8a	98.67 ± 1.3a

For each treatment, 20 cotyledonary leaf explants were used and maintained in three replicates; mean percentage of callus necrosis calculated after 4 weeks of incubation and the mean number of callus produced by each inoculated explant counted after 4 weeks of incubation in the dark. Values are mean ± SE. Means followed by the same letter are not significantly different at 0.05 % level based on Duncan’s Test

### Factors affecting transformation efficiency

#### Infection period

After 2–3 selection cycles at different lengths of the infection period (10, 15, and 20 min) but at the same concentration of bacterial cells (OD of 0.5), explant survival in AKS 207 was the highest (48.3 %) at 15 min, 33.3 % at 10 min, and 23.3 % at 20 min (Table 2). In PKV Pink too, the maximum survival (53.3 %) was recorded when the length of the infection period was 15 min (Table 2).

**Table 2:** Effect of different transformation parameters on explant survival and callus formation under selection pressure

Bacterial cell density	Infection period (min)	No of explants inoculated	No of callus survived AKS-207	No of callus survived PKV PINK	Callus formation of AKS-207(%)	Callus formation of PKV PINK (%)
Transformation conditions						
0.25	5	60	3	4	5.00 ± 2.9fg	6.67 ± 1.7hi
	10	60	5	5	8.33 ± 1.7efg	8.33 ± 4.4ghi
	15	60	11	9	18.33 ± 3.3cdefg	15.00 ± 2.9fgh
	20	60	6	7	10.00 ± 5.0defg	11.67 ± 1.7ghi
0.5	5	60	10	13	16.67 ± 4.4defg	21.67 ± 4.4def
	10	60	20	22	33.33 ± 3.3bc	36.67 ± 4.4b
	15	60	29	32	48.33 ± 6.0a	53.33 ± 7.3a
	20	60	14	11	23.33 ± 6.0cde	18.33 ± 4.4efg
0.75	5	60	13	14	21.67 ± 3.3cde	23.33 ± 9.3efg
	10	60	23	19	38.33 ± 8.8bcd	31.67 ± 6.0bc
	15	60	15	18	25.00 ± 5.8ab	30.00 ± 7.6cde
	20	60	11	10	18.33 ± 4.4bcd	16.67 ± 6.0efg
1	5	60	12	12	20.00 ± 5.8cdef	20.00 ± 2.9efg
	10	60	11	17	18.33 ± 4.4cdefg	28.33 ± 6.7bcd
	15	60	7	6	11.67 ± 4.4defg	10.00 ± 5.0hi
	20	60	2	1	3.33 ± 1.7g	1.67 ± 1.7i
BGM containing AS in µM						
LB	0	150	1	3	0.67 ± 0.7e	2.00 ± 1.2c
	50	150	15	10	10.00 ± 3.5de	6.67 ± 2.9c
	100	150	41	32	27.33 ± 6.6bc	21.33 ± 1.8b
	200	150	35	34	23.33 ± 4.8bcd	22.67 ± 2.9b
AB	0	150	21	3	14.00 ± 3.5cde	2.00 ± 1.2c
	50	150	29	27	19.33 ± 5.8bcd	18.00 ± 3.1b
	100	150	59	63	39.33 ± 2.9a	42.00 ± 4.0a
	200	150	36	42	24.00 ± 2.3bcd	28.00 ± 3.5b
MS	0	150	13	4	8.67 ± 2.9de	2.67 ± 1.8c
	50	150	35	36	23.33 ± 5.8bcd	24.00 ± 5.3b
	100	150	50	52	33.33 ± 3.5b	34.67 ± 5.5a
	200	150	40	36	26.67 ± 8.2bc	24.00 ± 6.4b
Co-cultivation duration hours						
	0	60	1	0	1.67 ± 1.7c	0.00 ± 0.0c
	24	60	26	23	43.33 ± 4.4b	38.33 ± 7.3b
	48	60	41	47	68.33 ± 10.9a	78.33 ± 6.0a
	72	60	13	13	21.67 ± 4.4c	21.67 ± 4.4b

Mean percentage of callus necrosis calculated after 4 weeks of incubation and the mean number of callus produced by each infected explant counted after 4 weeks of incubation in the dark. Values are mean ± SE. Means followed by the same letter are not significantly different at 0.05 % level based on Duncan’s test

#### Acetosyringone and bacterial growth medium

Explant survival under selection pressure was significantly affected by the BGM (Table 2), the survival percentage being significantly higher in calli infected with bacteria grown on the AB medium than that on MS or LB medium. After 4 weeks, the transformation frequency was significantly higher on AB medium containing 100 µM AS than that on AB medium without AS (Table 2). The transformation frequencies for AKS 207 and PKV Pink were 39.3 and 42.0 %, respectively, on AB medium, followed by 33.3 and 34.7 % on MS medium and 27.3 and 21.3 % on LB medium (Table S1).

#### Duration of co-cultivation

Transformation efficiency using *Lentil*-*lectin* gene construct was directly correlated with the duration of co-cultivation, increasing significantly with duration up to 48 h but declining thereafter. A longer co-cultivation period (72 h) led to bacterial contamination, and no healthy shoots could be recovered.

#### Genotype

**Fig. 3:**
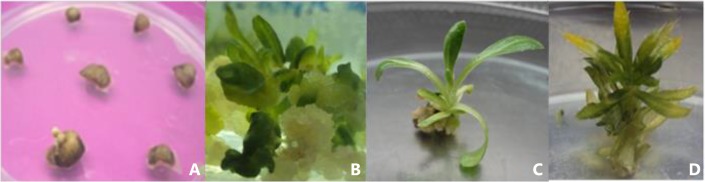
Different stages of putative transformed callus and in vitro shoot regeneration on the selection medium. **a** Callus induction from cotyledonary leaf explants after 3–4 weeks, **b** shoot induction after 4–5 weeks of Callus formation, **c** multiple shoot after 2–3 week of Shoot induction and **d** elongated shoots seen after 1–2 weeks of multiple shoot formation

Deploying the optimized transformation parameters mentioned above, a genotype-independent protocol was used for both genotypes, namely cotyledonary leaf explants infected with a diluted culture of *Agrobacterium* (0.5 OD) grown on AB medium supplemented with 100 µM AS. The explants thus raised were washed and transferred to the selection medium (kanamycin 50 mg/L and cefotaxime 250 mg/L). Callus formation was observed 4 weeks after transformation (Fig. 3a): the putatively transformed shoots developed on MS medium containing BAP—3 mg/L for AKS 207 and 4 mg/L for PKV Pink—in 4–5 weeks after callus formation (Fig. 3b), and further growth of putatively transformed multiple shoots was observed 2–3 weeks after shoot induction (Fig. 3c). The multiple shoots grew 1.5–2.5 cm in length after 1–2 weeks of their formation (Fig. 3d).

### Histochemical assay

**Fig. 4:**
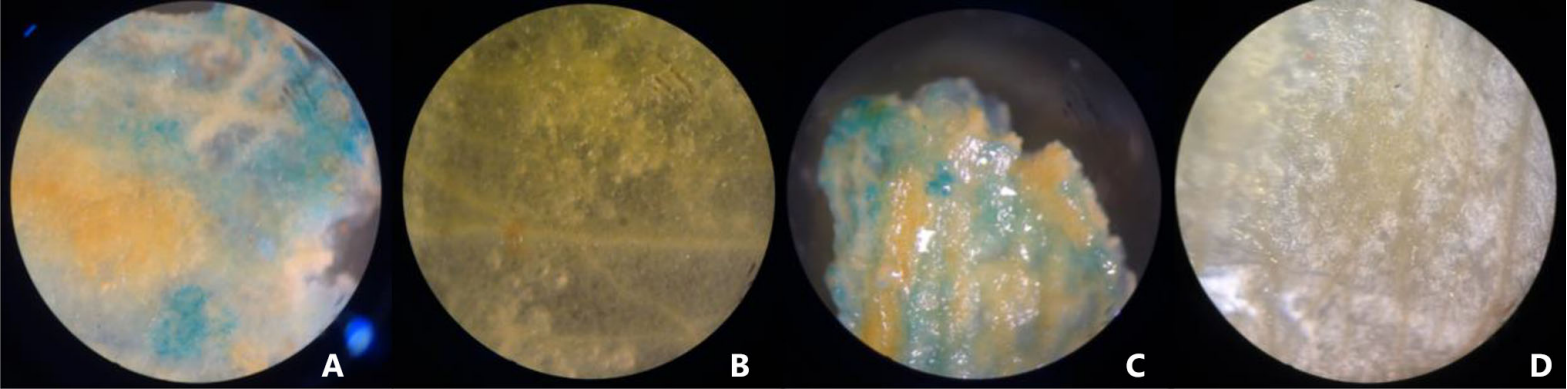
Screening of Putative transformed shoots of safflower through GUS assay observed under microscope by horizontal thin section. **a** putative transformed multiple shoot tissue of AKS-207 showing GUS expression, **b** non-transformed multiple shoot tissue of AKS-207 used as negative Control, **c** putative transformed multiple shoot tissue of PKV Pink showing GUS expression and **d** non-transformed multiple shoot tissue of PKV Pink

Primary transformants obtained after 5–7 cycles of selection from all the above experiments were used for GUS assay and non-transformed shoots were used as the negative control: GUS expression was ascertained by looking for blue spots (Fig. 4). A total 37 shoots of AKS 207 and 21 shoots of PKV Pink, each 1.5–2.5 cm long, were excised and used, of which 20 and 10, respectively, showed GUS expression, thus giving a transformation frequency of kanamycin-resistant shoots of 54.0 and 47.6 %, respectively (Table S1).

### Confirmation of GUS-positive shoots using polymerase chain reaction

**Fig. 5:**
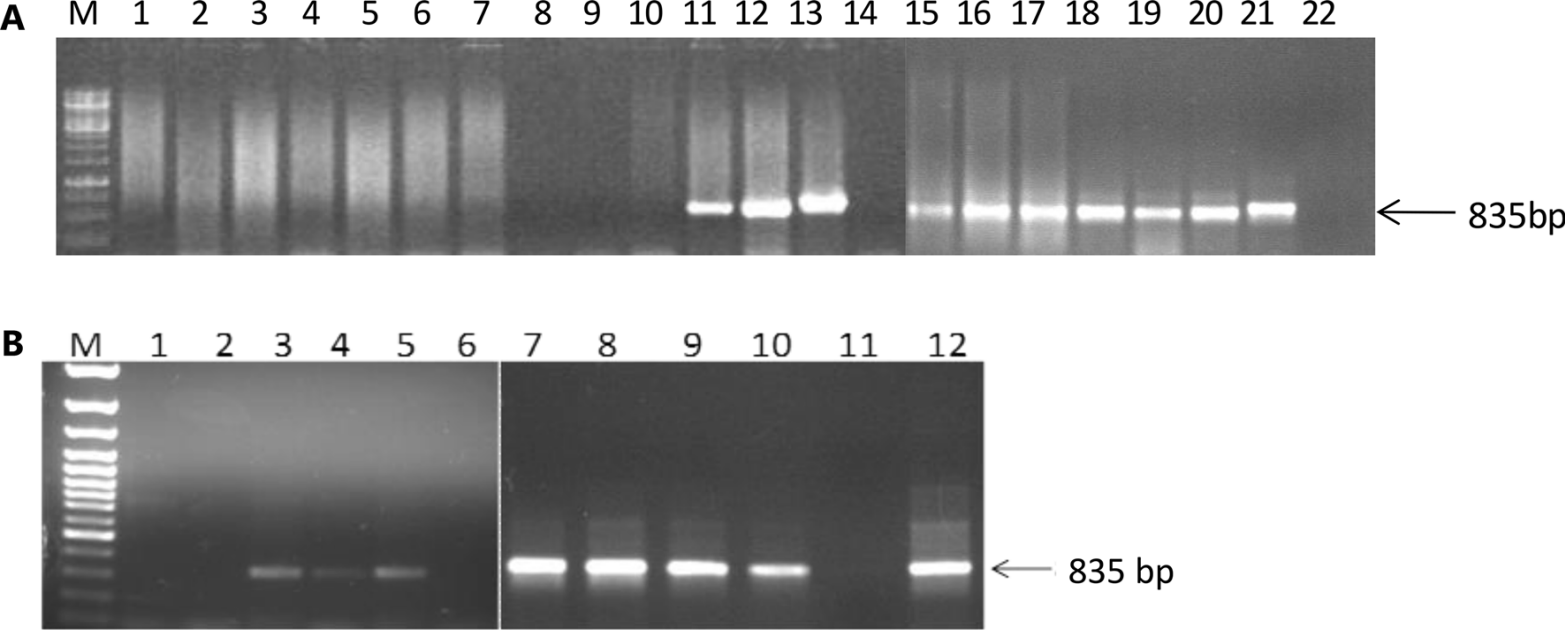
**a** PCR amplification of gDNA of GUS-positive AKS-207 putative transformed shoots. M-1 Kb DNA ladder, 1–20-GUS-positive plantlets, 21-positive control plasmid DNA and 22-negative control gDNA of untransformed PKV pink. **b** PCR amplification from gDNA of GUS-positive PKV Pink putative transformed shoots. M-1 Kb DNA ladder, 1-10-GUS-positive plantlets, 11-negative control gDNA of un-transformed PKV Pink and 12-positive control plasmid DNA

Genomic DNA of GUS-positive tissues of AKS 207 and PKV Pink was isolated and amplified using primers specific to the *Lentil*-*lectin* gene: ten multiple shoots of AKS 207 and seven of PKV Pink showed positive integration of the *Lentil*-*lectin* gene (Fig. 5a, b). Inline results were reported by Ying et al. (1992) for transformation of safflower calli. The frequency of transformation in GUS-positive plants through PCR analysis was 27.0 % for AKS 207 and 33.3 % for PKV Pink (Table S1). After 3–4 weeks, a total of 12 plants (eight putatively transformed and four controls) of AKS 207 and a total of eight plants (two and six) of PKV Pink showed very few roots on half-strength MS medium supplemented with NAA (2 mg/L). For hardening, these plants were transferred to pots filled with soil but, with such sparse rooting, none could survive (Fig. S3).

## Discussion

The safflower aphid (*Uroleucon compositae*) is the most serious pest of safflower; depending on the environmental conditions, yield losses can be 30–80 % (Hanumantharaya et al. 2008). Developing safflower cultivars resistant to the aphid through transgenic means is a more environment-friendly option than using non-biodegradable and persistent chemical insecticides. *Agrobacterium*-mediated transformation in safflower was limited only to selectable marker and reporter gene constructs (Ying et al. 1992; Rohini and Rao 2000; Belide et al. 2011; Motamedi et al. 2011; Sujatha et al. 2012). Insect-resistant transgenic crops have been produced using overexpression of *Bt* (*Bacillus thuringiensis*) toxins, but these toxins exhibit little toxicity against such homopteran insects as aphids, mealybugs, and whiteflies (Chougule and Bonning 2012; Shingote et al. 2013). We, therefore, opted for the more effective *Lentil*-*lectin* gene construct for transformation of safflower.

Selection and optimization of the selective medium with specific antibiotics are necessary for the growth of transformed tissue. We found that 50 mg/L of kanamycin (LD_50_) optimal for both the genotypes (50.0 % survival even after 4 weeks of the kanamycin selection cycle; Table 1). Motamedi et al. (2011) and Belide et al. (2011) used the same concentration of kanamycin for selecting transformed calli in safflower, and so did Venkatachalam et al. (1998) in groundnut and Yadav et al. (2010) in sesame. The bacteriostatic antibiotic cefotaxime at 250 mg/L was found effective for a safflower transformation protocol similar to that reported by Rao and Rohini (1999).

Both titre (strength of the suspension of *Agrobacterium* cells) and the length of the infection period affected the success of transformation significantly (Table 2). At low titres, the extent of colonization and the frequency of transformed shoots were also low; at the same time, very high titres (OD of 1.0) and prolonged exposure (20 min) to them also proved detrimental to transformation efficiency and led to greater bacterial contamination, probably because the explants were severely injured. The ideal combination comprised a titre of 0.5 (OD_600_) and 15 min of exposure. Similar results were reported by Shilpa et al. (2010) although Belide et al. (2011) found 0.4 OD to be optimal and Sujatha et al. (2012) found 0.6 OD to be optimal for transformation.

The influence of AS on T-DNA transfer was tested in terms of the survival percentage of calli after co-cultivation on kanamycin (50 mg/L) (Fig. 3b). A combination of 100 µM of AS and AB medium was found optimum, leading to as many as 39–42 % of the explants of both genotypes forming calli (Table 2). Further increase in AS concentration above 100–200 µM resulted in reduced transformation efficiency for both genotypes.

Transferring the explants directly onto the selection medium drastically lowered the transformation efficiency whereas extending the period of co-cultivation up to 48 h increased the transformation efficiency significantly. Co-cultivation beyond 48 h, however, resulted in excessive bacterial growth and lowered the efficiency. Similar results have been reported by several researchers (Muthukumar et al. 1996; Sujatha et al. 2012).

The frequency of transformation was 27.0 % in AKS 207 and 33.3 % in PKV Pink (Table S1). Earlier researchers reported very low regeneration frequency, which is fully dependent on the genotype, source and age of explants, and composition of the growth medium (Mandal and Gupta 2001; Radhika et al. 2006). We also studied the effect of different concentrations of hormones, but were unable to harden the regenerated and putatively transformed shoots sufficiently for them to survive the transfer to soil. Shoot regeneration frequency is very high but needs to be converted to genetic transformation frequencies for successful development of transgenic plants. In safflower, rooting frequency was very low in our earlier study (Dhumale et al. 2015) and in other studies involving other varieties of safflower (Nikam and Shitole 1999; Sujatha 2007, Belide et al. 2011). Poor rooting is a critical setback and must be overcome for successful application of transgenic methods in safflower.

## Electronic supplementary material

Below is the link to the electronic supplementary material.

Supplementary material 1 (DOCX 22136 kb)
