# Supplementary material for: Parameters influencing Agrobacterium-mediated transformation system in safflower genotypes AKS-207 and PKV Pink
Source: 3 Biotech. 2016 Aug 26;6(2):181. doi: 10.1007/s13205-016-0497-4 (PMC5001957; doi:10.1007/s13205-016-0497-4)
Supplement: Supplementary file 1 — Supplementary material 1 (DOCX 22136 kb) [file 13205_2016_497_MOESM1_ESM.docx]

**Fig S1.** The callusing of inoculated explants on MS medium supplemented with 2, 4-D and KIN in the ratio of A. 1:1, B. 1:2 and C. 1:3 (1mg/L each, 1 & 2 mg/L, and 1 & 3 mg/L 2, 4-D and KIN respectively).

**Fig S2.** Multiple Shoot formation on MS media containing different BAP concentration of AKS-207 and PKV Pink genotype safflower genotypes. A. BAP (1mg/L), B. BAP (2mg/L), C. BAP (3mg/L), D. BAP (4mg/L) & E. BAP (5mg/L).

**Fig S3.** Hardening of putatively transformed and non-transformed tissue culture raised safflower genotypes A. Non-transformed safflower plants, B. putative transformed plants of AKS-207 and C. putative transformed plants of PKV Pink.

**Table S1.** Frequency of transformation after GUS assay and PCR analysis

| **Varieties** | **No. Of shoots regenerated on selection medium** | **No. of GUS positive plants** | **Frequency of transformation for GUS expressing shoots** | **No of PCR positive plants** | **Transformation efficiency** |
| --- | --- | --- | --- | --- | --- |
| **AKS-207** | 37 | 20 | 54.05% | 10 | 27.02% |
| **PKV pink** | 21 | 10 | 47.61% | 7 | 33.33% |
